# Opposing Functions of the ETS Factor Family Define *Shh* Spatial Expression in Limb Buds and Underlie Polydactyly

**DOI:** 10.1016/j.devcel.2011.12.010

**Published:** 2012-02-14

**Authors:** Laura A. Lettice, Iain Williamson, John H. Wiltshire, Silvia Peluso, Paul S. Devenney, Alison E. Hill, Abdelkader Essafi, James Hagman, Richard Mort, Graeme Grimes, Carlo L. DeAngelis, Robert E. Hill

**Affiliations:** 1 MRC Human Genetics Unit, MRC Institute of Genetics and Molecular Medicine, University of Edinburgh, Western General Hospital, Crewe Road, Edinburgh EH4 2XU, UK; 2 Integrated Department of Immunology, National Jewish Health, Denver, CO 80206, USA

## Abstract

Sonic hedgehog (*Shh*) expression during limb development is crucial for specifying the identity and number of digits. The spatial pattern of *Shh* expression is restricted to a region called the zone of polarizing activity (ZPA), and this expression is controlled from a long distance by the *cis*-regulator ZRS. Here, members of two groups of ETS transcription factors are shown to act directly at the ZRS mediating a differential effect on *Shh,* defining its spatial expression pattern. Occupancy at multiple GABPα/ETS1 sites regulates the position of the ZPA boundary, whereas ETV4/ETV5 binding restricts expression outside the ZPA. The ETS gene family is therefore attributed with specifying the boundaries of the classical ZPA. Two point mutations within the ZRS change the profile of ETS binding and activate *Shh* expression at an ectopic site in the limb bud. These molecular changes define a pathogenetic mechanism that leads to preaxial polydactyly (PPD).

## Graphical abstract

**Graphical Abstract:**

**Figure:**
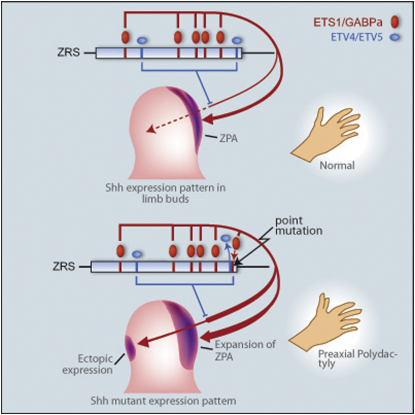

**Highlights:**

► ETS factors bind to the *Shh* enhancer, ZRS, to specify spatial expression in the limb ► ETS1 and GABPα bind to multiple sites to define the expression domain boundary ► ETV4 and ETV5 bind to inhibit expression outside that spatial domain ► Mutations that change the ETS binding profile cause limb abnormalities

## Teaser

A long-range limb-specific enhancer controls *Sonic hedgehog* expression in a spatiotemporally restricted manner. Lettice et al. identify ETS-binding sites within this enhancer that mediate antagonistic interactions between different ETS transcription factor clades. Mutations in the enhancer that change the balance of power between ETS factors cause limb abnormalities.

## Introduction

The zone of polarizing activity (ZPA) was experimentally defined as the region located at the posterior margin of the developing limb bud that determines digit number and identity (Hill, 2007; Tickle, 2006). The polarizing activities attributed to the ZPA are mediated by sonic hedgehog (SHH) which is postulated to act as a diffusible morphogen. A number of models have been proposed to explain *Shh* activity (Towers and Tickle, 2009) and most recently, SHH was shown to act as both a morphogen and a mitogen to coordinate digit formation by integrating growth with digit specification during limb development (Towers et al., 2008; Zhu et al., 2008). The restricted spatial expression within the ZPA is an essential element of all the proposed models of *Shh* function (Ahn and Joyner, 2004; Harfe et al., 2004; Yang et al., 1997).

A critical step in understanding the complexity of *Shh* expression was the identification of the *cis*-regulatory element called the ZRS for *Z*PA regulatory *s*equence (Lettice et al., 2002, 2003) (also called MFCS1 [Sagai et al., 2005]). The ZRS comprises ∼800 bp of a multispecies conserved sequence and encodes most, if not all, of the information that regulates the spatiotemporal pattern of *Shh* expression in the developing limb bud. The ZRS is the paradigm for long-range gene regulation, operating over ∼1 Mb to regulate *Shh* (Lettice et al., 2003; Sagai et al., 2005). Single point mutations in the human ZRS are found in patients presenting with a range of limb skeletal malformations. These include preaxial polydactyly type 2 (PPD2), triphalangeal thumb polysyndactyly (TPTPS), syndactyly type IV (SD4), and Werner's mesomelic syndrome (WMS), collectively referred to as ZRS-associated syndromes (Lettice et al., 2003, 2008; Farooq et al., 2010; Furniss et al., 2008; Gurnett et al., 2007; Semerci et al., 2009; Wieczorek et al., 2010). The point mutations function to generate ectopic expression at the anterior margin of the limb bud (Furniss et al., 2008; Lettice et al., 2008), which is the underlying cause of PPD.

Here, members of the large group of ETS transcription factors (Sharrocks, 2001) are shown to play distinct roles in the spatial pattern of *Shh*. Occupancy at multiple ETS sites, which bind the factors GABPα and ETS1, regulates the position of the *Shh* expression boundary in the limb, thus defining the ZPA. Multiple binding of ETV4 and ETV5 at the ZRS, in contrast, represses ectopic *Shh* expression outside the ZPA. In addition, we show that two PPD mutations disrupt the balance in number of ETS binding sites derepressing expression in the anterior limb bud.

## Results

### Multiple ETS Sites Identified in the ZRS

**Figure 1:**
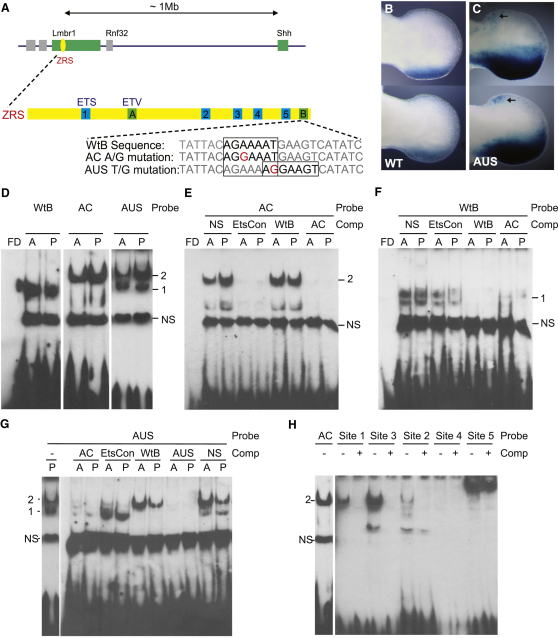
Point Mutations Alter *Shh* Expression and Protein Binding Profiles (A) Schematic showing the ZRS (yellow box), which resides within intron 5 of LMBR1, 1 Mb for the *Shh* gene. The positions of the ETS sites 1–5 and ETV sites A and B identified within the ZRS are marked by blue and green boxes, respectively. The sequences around the mutations identified in families with PPD (Family AC and AUS) are shown. (B and C) Limbs from transgenic animals carrying wild-type (B) and mutant (C) ZRS reporter constructs (forelimb buds are shown on top and hindlimb buds below) demonstrate that the AUS mutation results in expansion of the posterior expression (compare to B) and ectopic staining in the anterior mesenchyme (arrows). (D–H) EMSA analysis of nuclear extracts from anterior (A) and posterior (P) halves of E11.5 limb buds. (D) Nuclear extract was incubated with ds-oligos containing the WT, AC, or AUS sequence. The WT sequence produced a specific band (1); the AC point mutation resulted in a higher migrating band (2); and the AUS mutation produced a combination of WT and AC binding; bands 1 and 2. A nonspecific (NS) band was observed for all ds-oligos. (E–G) EMSA using the AC ds-oligo (E), WT ds-oligo (F), and AUS ds-oligo (G), and using an unlabeled NS sequence, ETS consensus sequence (EtsCon), WT, or AC oligonucleotide as their competitors. (H) Comparison by EMSA of the binding for the wild-type ZRS sites 1–5, showing a greater extent of binding to the AC mutant site and sites 1 and 3. The unlabelled AC oligonucleotide (lanes labeled +) specifically competes for band 2.

The notion of “homotypic clustering” (Gotea et al., 2010; Lifanov et al., 2003; Wagner, 1999) suggests that *cis*-regulators contain multiple sites for crucial regulatory factors. Analysis of the ZRS identified a 7 bp motif (AGGAA^G^/_A_T) that is repeated five times (Figure 1A), with each repetition located in a highly conserved position (Figure S1A available online). This purine-rich sequence is contained within the consensus for the 8 bp ETS1 binding motif (^C^/G/_A_AGGAA^G^/_A_T) found in putative distal regulators of genes in T cells (Hollenhorst et al., 2009). None of the numerous point mutations in the ZRS that cause limb deformities fall within these conserved motifs; however, mutations identified in two families with PPD2, Family A & C (AC) (Gurnett et al., 2007) and an Australian family (AUS) (E. de Graaff, personal communication), convert the surrounding sequence to additional ETS motifs (Figure 1A).

Transgenic analysis using a construct containing the ZRS drives expression of the LacZ reporter gene in the expected posterior position in the limb (Furniss et al., 2008; Lettice et al., 2003, 2008) (Figure 1B). Addition of the AUS point mutation to the ZRS was sufficient to drive the ectopic expression at the anterior margin of E11.5 limb buds (Figure 1C, arrows). (The number of transgenic embryos is reported in Table S1.) The mutation also caused an overall increase in the width of posterior expression such that the boundary extended deeper into the middle of the limb (Figure 1C).

### Differential Binding at the Mutant and Wild-Type Sites of the ZRS

The protein-binding profile associated with the 7 bp motif was analyzed by electrophoretic mobility shift assays (EMSAs) using biotin-labeled double-strand oligonucleotide (ds-oligos) probes and nuclear extracts from E11.5 embryonic limbs. Initially, the AUS and AC mutant sites were analyzed using a series of 24 bp ds-oligos (Figure 1A) spanning either the wild-type sequence (WtB) or the mutant sequences. The WtB sequence probe produced a single specific band (band1 in Figure 1D), which was replaced by a higher migrating band in the presence of the AC mutation (band 2 in Figure 1D). In comparison, the AUS mutant probe exhibited a combination of both the WtB and AC mutant band-shift patterns. Specificity of binding was confirmed using unlabelled competitors for either the WtB or the AC mutant sequence (Figures 1E–1G). An unrelated sequence (Fisher et al., 1992) that contained the 8 bp ETS1 CAGGAAGT motif (designated EtsCon) competed for the upper band detected by both the AC (Figure 1E) and AUS (Figure S1B) probes; whereas, the WtB probe competed but with an appreciably lower affinity (Figure 1F). In contrast, competition with the unlabelled WtB sequence did not affect the AC banding pattern (Figure 1E) but did disrupt binding of the lower band detected with the AUS sequence (Figure 1G). These data are consistent with the two point mutations binding similar proteins (Figure 1A) at closely apposed positions. The two mutations affect wild-type protein binding differently; whereas the AC mutation causes replacement of wild-type binding, the AUS mutation allows binding of either protein.

The five endogenous AGGAA^G^/_A_T sites within the 800bp ZRS (Figure S1A) were also analyzed. ds-Oligo probes for sites 1, 2 and 3 each generated a band that migrated to the same position as that for the AC probe (Figure 1H) and showed specificity for binding by competition with the unlabelled AC probe. Site 4 probe did not detectably bind a protein while site 5 bound an unidentified, higher migrating band which was not competed with the AC sequence.

### Identification of the Factors that Bind the ZRS In Vivo

**Figure 2:**
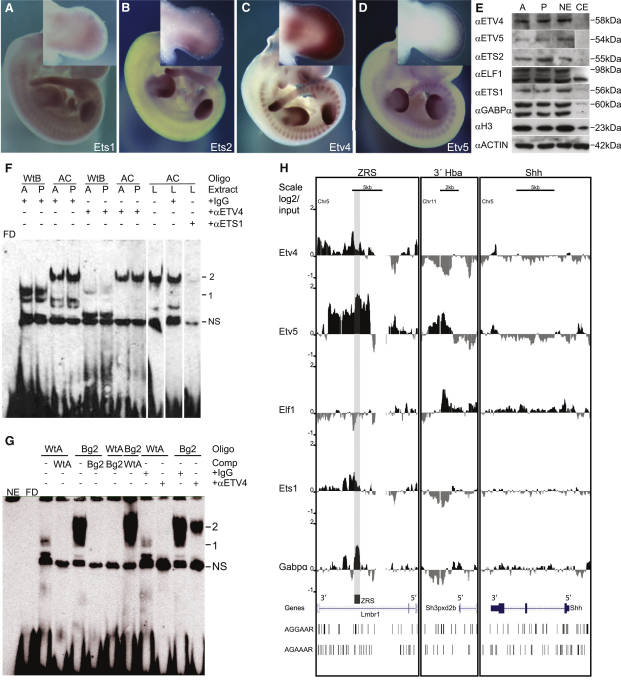
ETS Factors Are Expressed in the Limb and Bind to the ZRS (A–D) Whole-mount in situ hybridization analysis for *Ets1* (A), *Ets2* (B), *Etv4* (C), and *Etv5* (D) are shown in E11.5 embryos and limb buds. (E) Western blot analysis using antibodies raised against ETS factors, designated αETV4, αETV5, αETS2, αELF1, αETS1, and αGABPα, and against histone H3 (αH3) and actin (αactin), with nuclear extracts from the anterior (A) and posterior (P) halves of the limb buds (E11.5). Also shown is a comparison between limb nuclear extracts (NE) and cytoplasmic extracts (CE). αH3 and αactin were used as loading controls. (F) EMSA shows WtB and AC ds-oligo binding in nuclear extracts depleted for ETV4 or ETS1 using specific antibodies (αEtv4 and αEts1). (IgG was used as a nonspecific control.) Extracts from anterior (A) or posterior (P) halves or whole limbs (L) from E11.5 limb buds were used. Band 1 observed with the WtB probe was specifically depleted by the addition of αETV4 antibody, while Band shift 2 observed with the AC probe was specifically depleted by the addition of αETS1 antibody. (G) EMSAs were conducted with ds-oligos containing the sequence for the wild-type ETV4 site A (WtA) or the Belg2 mutation (Bg). WtA ds-oligo shows a specific band (1) while that for Bg sequence gives an additional higher migrating band (2). The anti-ETV4 antibody depletes Band 1 observed with WtA and Bg probes (nonspecific IgG used as control). (H) ChIP using antibodies to five different ETS factors (ETV4, ETV5, Elf1, ETS1, and GABPα) analyzed by hybridizing to tiling microarrays. Summary is presented using three different genomic regions, the y axis is Log_2_ for each ChIP/input DNA and the x axis represents a segment of DNA from the microarray. The DNA region containing the ZRS is highlighted by the gray shading. As controls, the whole of the *Shh* coding region plus promoter (Shh) and the region downstream of the α-globin locus (3′Hba) are shown. Scale bars are shown at the top and the positions of potential ETS1/GABPα (AGGAA^G^/_A_) and ETV4/ETV5 (AGAAA^G^/_A_) binding sites at the bottom of each panel.

A number of ETS family members are expressed in the early-stage limb and are distributed along the distal mesenchyme. *Ets1* and *Ets2* (Ristevski et al., 2002) (Figures 2A and 2B) are expressed in the posterior mesenchyme overlapping the *Shh* domain in the limb bud at E10.5 and by E11.5 extend distally, incorporating the anterior margin (Figure 2A). *Etv4* and *Etv5* (also called *Pea3* and *Erm*, respectively) (Figure 2C and 2D) (Mao et al., 2009; Zhang et al., 2009) are expressed in the mesenchyme along the entire distal edge. *Gabpα* (EMAGE:2836) (Ristevski et al., 2004) is reportedly more highly expressed in the distal mesenchyme in the posterior margin of the limb and *Elf1* (EMAGE:1462) is expressed throughout the entire limb mesenchyme (Richardson et al., 2010).

To investigate the binding of candidate ETS proteins to the ZRS sequences, we used ETS antibodies specific for ETS1, ETS2, GABPα, ELF1, ETV4, and ETV5. Western blot analysis of limb extracts (Figure 2E) showed that these proteins are produced in both anterior and posterior halves of the limb bud and are enriched in the nuclear extracts. EMSA analysis showed that the anti-ETV4 (αETV4 in Figure 2F) antibody depleted the binding of the wild-type protein responsible for the WtB banding pattern, whereas the anti-ETS1 antibody (αETS1 in Figure 2F) depleted the protein binding to the upper band of the AC sequence. The other antibodies did not detectably affect the banding pattern (data not shown).

The sequence that ETV4 binds encompasses the noncanonical site AGAAAAT (referred to as ETV4 site B) (Figures 1A and S2A) (Xin et al., 1992). A second ETV4 binding site found to contain the AGAAA sequence (referred to as site A) (Figures 1A and S1) is the site of the previously published PPD2 mutation Belg2 (Lettice et al., 2003). The Belg2 mutation, which converts the sequence to AGGAA, was previously shown to drive ectopic expression in transgenic assays (Lettice et al., 2008). EMSA demonstrated that ETV4 binds the Belg2 wild-type ds-oligo (WtA), as confirmed by depletion with the ETV4 antibody (αETV4 in Figure 2G). Analysis of the mutant Belg2 sequence, however, showed binding to an additional unidentified factor (band 2 in Figure 2G).

To establish that ETS factors bind directly to the ZRS in the limb, we performed chromatin immunoprecipitation (ChIP) in nuclear extracts from whole autopods of E11.5 embryonic limbs with the series of antibodies above. GABPα and ELF1 were of particular interest, given that recent analysis of ETS1 binding in vivo showed co-occupation by these factors at a substantial subset of sites (Hollenhorst et al., 2007, 2009). In our initial screen of the ChIP, neither the ETS2 nor the ELF1 antibody showed any appreciable binding to the ZRS using qPCR (data not shown). Further analysis using high-density tiling microarrays showed the binding of both ETS1 and GABPα to the ZRS region (Figure 2H). The binding profiles suggested that GABPα occupied the whole region of the ZRS, whereas ETS1 binding overlapped but was skewed toward the 3′ end of the ZRS. Accordingly, the two sites at the 3′ end (sites 4 and 5) contain the sequence AGGAA**A**T (Figure S1A), while the remaining sites contain AGGAA**G**T. The *Shh* gene (Figure 2H), including the promoter, and other control regions showed no binding to any of these ETS factors. ELF1 was used as a negative control and showed no binding (Figure 2H) at the ZRS, while appreciable binding was detected at a clustering of AGGAAA sites located near the 5′ end of an uncharacterized SH3-containing gene (3′Hba, Figure 2H).

ETV4 and ETV5 act to repress *Shh* expression in the posterior margin of the limb bud to ensure that no ectopic production occurs (Mao et al., 2009; Zhang et al., 2009). In limb extracts, both the anti-ETV4 and the anti-ETV5 antibodies detect binding to the ZRS region (Figure 2H). ETV4 binding appeared as two peaks in the region of the ZRS, which reflects the location of the two predicted ETV4 sites. Surprisingly, ETV5 occupies a broad region of >5 kb with a peak of binding over the whole of the ZRS (Figure 2H). ETV5 binding is highly specific for the ZRS region, as shown by the tiling microarray over the whole 1 Mb of the *Shh* locus (Figure S2B). ETV5 binds the ZRS directly; the broad region may reflect the high density of AGAAA^G^/_A_ sites available at nearby sites (Figure 2H).

*Ets1* is expressed not only in the posterior mesenchyme but also along the anterior margin overlapping the ectopic domain driven by the AUS mutation (Figure 1C). To determine whether ETS1 binds to the ZRS at both the anterior and the posterior halves, we dissected limb buds at E11.5 for ChIP. qPCR showed significant binding of ETS1 at the ZRS (Figure S2C) in both the anterior and posterior halves, suggesting that the ZRS is open for factor interactions on the quiescent as well as the active side of the limb.

### Ectopic *Shh* Expression Occurs by Two Mechanisms

**Figure 3:**
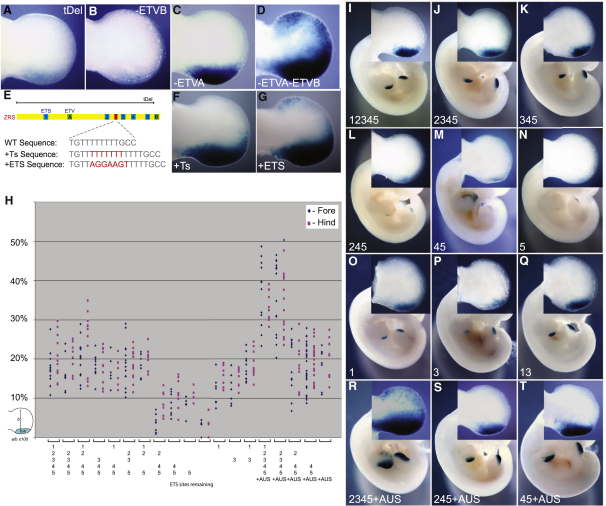
Transgenic Analysis of Embryos Carrying Mutant ZRS Sequences (A–D) Limbs from transgenic embryos carrying the following mutant ZRS sequences: the 44 bp terminal deletion (tDel) (A), the 3 bp change in the ETV4 Site B (-ETVB) (B), and Site A (-ETVA) (C). Disruption of both sites in combination (D) results in ectopic expression in the anterior of the limb. (E–G) Position of the run of Ts within the ZRS and the changes added are shown in red. Expression due to these changes is shown by comparison of the addition of seven Ts (F) and the extra ETS1/GABPα site (AGGAAGT) (G). Ectopic expression is detected in (G). (H) Graphical representation of the expression pattern resulting from mutations within the endogenous ETS sites. Expression pattern is the ratio of the width of the expression domain divided by the width of the limb, expressed as a percentage (see p values in Table S2). (I–Q) Examples of transgenic embryos for the ETS mutations analyzed are shown. The ETS sites remaining are depicted in the lower-left-hand corner of each figure. (R–T) Transgenic embryos that represent the addition of the AUS mutation in combination with ETS site mutations are shown. A close-up of a forelimb for each is shown in the insets.

We investigated two possible explanations for the ectopic expression driven by the AUS point mutation. First, by displacing the ETV4/ETV5 repressor at site B (Figure 1A), the binding of GABPα/ETS1 to the mutant site may lead to ectopic activation. To investigate this possibility, we generated two different mutations that disrupt site B. Neither a terminal deletion of 44 bp (tDel, Figure 3A), which removes the entire site, nor replacement of three base pairs within the ETV4 binding site (AGA**AAA**T→AGA**GCG**T) (-ETVB, Figure 3B) caused ectopic expression in transgenic embryos. In fact, expression levels decreased but importantly, the spatial boundary appears unaffected. Thus we tested a second possibility, specifically that the AUS mutation, by creating an additional AGGAAGT site, may directly drive ectopic activity. An extra AGGAAGT site was added to the ZRS. A putative neutral position, a variable stretch of Ts that differ in mammalian species (human has six Ts, mouse has eight, and rat has 22), was selected as the site for the insertion (Figure 3E). The modified ZRS generated ectopic expression activity (Figure 3G), whereas the control (insertion of seven Ts [Figure 3E]) showed no detectable ectopic expression (Figure 3F). These data show that an additional GABPα/ETS1 site is sufficient, on its own, to generate ectopic expression.

These analyses, as a consequence, raised questions about the direct role ETV4/ETV5 plays in restricting ectopic expression of *Shh*. To investigate the regulatory role of the ETV4/ETV5 proteins further, the three-base-pair replacement in site B, discussed above, was made in site A. Similar to the case with loss of only site B, no ectopic expression was observed with loss of site A (Figure 3C). The double mutation of both sites A and B, however, resulted in ectopic expression along the distal, anterior margin of the limb (Figure 3D). The simultaneous removal created a loss of ETV function and confirms that this ETV subfamily acts directly at the ZRS to restrict the expression pattern of *Shh* to the posterior margin.

### The Endogenous GABPα/ETS1 Sites Define the Boundary of *Shh* Expression

To investigate the consequences of having multiple, clustered ETS sites, the five endogenous sites in the ZRS were systematically inactivated (**AGG**AA^G^/_A_T → **CTT**AA^G^/_A_T) and evaluated by the transgenic assay (Figures 3H–3Q). For each transgenic, the relative domain widths of the limb buds were measured as indicated in Figure 3H and plotted. The mean domain widths of fore- and hindlimbs combined for each experimental group were then compared. The mean size of the expression domain was found to be highly significantly related to the combination of ETS sites that were disrupted (analysis of variance [ANOVA] p < 0.0001). The results of subsequent pairwise comparisons (Tukey's HSD test) are shown in Table S2.

Disruption of both sites 1 and 3 (the two sites that showed highest affinity for ETS1 binding in vitro) (Figure 3L) resulted in a significant reduction (p values in Table S2) in reporter-gene expression as compared to the wild-type construct (Figure 3I), whereas singly (Figure 3J), neither site detectably changed limb-bud expression (Figures 3I–3K). Disruption of additional sites resulted in no further reduction in the expression domain (Figure 3H). The activity levels of sites 1 and 3 were further analyzed. The sole presence of either one of these sites (Figures 3O and 3P) established boundaries of expression approaching that of the wild-type construct, whereas the presence of both 1 and 3 together (Figure 3Q) generated a wide expression domain that was indistinguishable. Sites 2, 4, and 5 (Figure 3L) showed some activity, as in the presence of either site 1 or 3, producing an expression domain similar to that of the wild-type (Figure 3H).

The addition of the extra AUS mutant site caused a further upregulation in transgenic embryos, with expression extending deeper into the center of the limb (Figure 3H). The construct carrying the AUS mutation but lacking site 1 showed little change (Figure 3R), whereas, again, removing sites 1 and 3 caused a retraction of the expression boundary (Figure 3S) but only to the extent of that generated by the wild-type construct (Figure 3H). Further removal, deleting sites 1, 2 and 3 together (Figure 3T), or all five sites, caused little further change in expression, suggesting that this single high-affinity site is sufficient for generating the wild-type transgenic expression. With the inclusion of the AUS mutation, ectopic anterior expression occurs in the limbs (Figure 1C); however, in the absence of both sites 1 and 3 the number of limbs with ectopic expression decreases from 83% to 33%, and in the absence of all five sites (Table S1), no ectopic expression was detected, suggesting that the wild-type ETS sites assist in the ectopic expression. In addition, an earlier stage of limb development was examined (E10.5) using constructs containing the wild-type ZRS, and with site 1 and sites 1 and 3 disrupted and with the AUS mutation added. The relative size differences of the expression domains were unchanged; thus, no temporal differences were apparent (data not shown).

## Discussion

Asymmetric expression is essential for SHH morphogen activity in the developing limb. The ETS gene family plays a central role in the *Shh* spatial pattern, both positioning the expression of Shh at the posterior margin of the limb and repressing ectopic expression at the anterior margin. The limitation of the cell-free approach to identify the full spectrum of ETS family members was highlighted by the in vivo ChIP analysis, which identified additional binding factors. The co-occupancy identified for ETS1 and GABPα at the ZRS corresponds to the genome-wide occupancy analysis of Hollenhorst et al. (2009), which shows that the vast majority of GABPα sites colocalize with those that bind ETS1 sites. In addition, the ChIP approach showed that both ETV4 and ETV5 act directly at the ZRS. Even with the resolution afforded by ChIP in combination with tiling microarrays, it is difficult to determine whether GABPα and ETS1 or ETV4 and ETV5 are competing for the same sites; however, the differences in profiles do suggest that there are site preferences.

**Figure 4:**
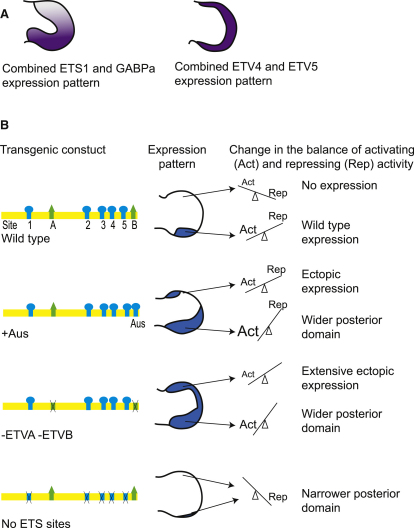
A Model Representing the Fine Balance of ETS Factor Binding and Their Effects on *Shh* Expression (A) Representation of the expression patterns of the activating ETS factors (ETS1 and GABPα) and the repressing ETS factors (ETV4 and ETV5). (B) A summary diagram of how four of the transgenic constructs are proposed to interact with the available ETS factors in the limb, with the expression pattern observed for each construct shown in the middle. The change in the balance between activating and repressing activity represented on the right shows the relative balance in the anterior and posterior margins of the limb. The size of the lettering represents the relative amounts of the activating and repressing activities.

Redundancy within this large group of ETS factors complicates both the biochemical and genetic analysis of gene function of individual family members. However, sequential inactivation of the ETS sites provided an approach to investigate the role these factors play. ETS1/GABPα directs the position of the *Shh* expression boundary delineating the experimentally defined ZPA. Our data suggest that *Shh* expression is most substantially affected by either site 1 or 3, and it appears that the two sites act cumulatively to achieve the wild-type position of the spatial boundary. Clearly, other sites participate given that, for example, the boundary produced by site 1 is augmented by the presence of sites 2, 4, and 5. The five binding sites do not participate equivalently, and affinity of binding, as detected in the EMSA, is probably part of the explanation. The combinatorial nature of these sites is further supported by the addition of the AUS mutant site in the ZRS, which causes extension of the expression boundary further into the middle of the limb bud. This mutant binding site is the most active of the sites analyzed and, on its own, is capable of driving expression to the approximate boundary of that generated by the wild-type construct. ETS1 and GABPα are expressed at highest levels in the posterior domain of the limb. We suggest that the levels of ETS1/GABPα and the multiple binding sites act in concert to establish a regulatory balance at the ZRS to, first, adjust the position of the *Shh* boundary and, second, in combination with the repressive activity of ETV4/ETV5, to restrict expression to the ZRS (see summary in Figure 4).

Two ETV4/ETV5 binding sites were identified in the ZRS. In transgenics, a single ETV binding site is sufficient to repress ectopic expression; the loss of both sites results in the loss of repressor activity and as a consequence activation of ectopic expression. Previously, expression of both ETV4 and ETV5 in the distal mesenchyme of the limb bud was shown to be maintained by FGF signaling emanating from the AER (Mao et al., 2009; Zhang et al., 2009). FGF is known to be essential for limb outgrowth and maintenance of *Shh* expression. We show that ETV4/ETV5 binding links FGF signaling directly to regulation at the ZRS, showing an unexpected role for FGF, acting through these factors, to repress expression at the anterior margin of the limb.

The AUS mutation has an additional pathogenetic activity, which is to drive ectopic expression in the developing limb bud. We show that the wild-type sites contribute to the ectopic expression but are dependent on the additional activity provided by the extra binding site. Previous data suggest that the ZRS is primed for activity in both the anterior and posterior margins of the limb bud (Amano et al., 2009). In accord, we show that the ZRS is open and fully capable of binding to activating factors such as ETS1 in the anterior domain of the limb bud. As a result, a new, mutant site such as that produced by the AUS mutation would be capable of binding ETS factors at both the normal and the ectopic domains of expression. ETV4/ETV5 is crucial for ensuring that at the primed ZRS, ectopic anterior expression does not occur during limb development (Figure 4). The addition of an extra single high-affinity ETS binding site (as with the AUS mutation) apparently overrides ETV4/ETV5 repression, causing the loss of *Shh* spatial restriction. These molecular events lead to the ectopic expression of *Shh* that underlies the preaxial polydactyly caused by the AC and AUS mutations.

The high conservation throughout the ∼770 bp of the ZRS suggests that there is scope for binding a complex mixture of factors. The ETS factor binding sites are most likely functioning along with the binding of other factors at the ZRS. In combination, these proteins would endow the ZRS with the properties that would not only delineate the boundary but also dictate precise temporal activity. In addition, multiple ETS sites along with other factors may encode an activity that is sufficiently robust to enable long-range recognition and activation of the *Shh* promoter.

## Experimental Procedures

### Materials

The antibodies used were: IgG (Santa Cruz, sc-2025), ETS1 (Maier et al., 2003), ETS2 (a kind gift from R. M. Roberts), ETV4 (Abcam, ab860902), ETV5 (Abcam, ab102010), GABPα (Santa Cruz, sc-22810), and ELF1 (Santa Cruz, sc-631).

### Transgenic Assay and In Situ Hybridization

Transgenic embryos were made and stained in accordance with standard techniques (Lettice et al., 2003), and assembly of mutant ZRS constructs is described in the Supplemental Information. Whole-mount in situ hybridizations were described previously (Hecksher-Sørensen et al., 1998). The *Etv4*, *Etv5*, and *Ets2* probes were transcribed from EST cDNA clones (Geneservice), whereas the *Ets1* probe was generated by RT-PCR and cloned into pZero (Invitrogen). Primers used to amplify Ets1 were 5′-GGAGCATCTAGAGATCCTGC-3′ and 5′-CAGCCATCTCCTGTCCAGC-3′.

### Measuring the Depth of ZRS Staining and Statistical Analysis

Measurement of the extent of expression in each transgenic in the ZRS shown in Figure 3H was measured in Photoshop and calculated as a percentage of the width of the limb bud (to correct for stage differences between the embryos), as shown in Figure 3H.

Statistical comparisons were performed using the statistical package R (http://www.r-project.org/) (R Development Core Team, 2008). For these comparisons, the hind- and forelimb data for each injected construct were combined and a one-way analysis of variance (ANOVA) was used to compare the mean values between each of the 19 groups. The result was highly statistically significant. Therefore, further pairwise post-hoc tests were performed to compare the individual groups using Tukey's HSD (honestly significant difference) test. The table of significance values is found in Table S2.

### Electrophoretic Mobility Shift Assays

Nuclear extracts were prepared directly from embryonic limb tissue (E11.5). EMSA analysis is described in detail in the Supplemental Information.

### Chromatin Immunoprecipitation and Tiling Microarrays

Cells from dissected E11.5 limbs were fixed with 1% formaldehyde (25°C, 10 min) and stopped with 0.125 M glycine. Crosslinked ChIP was performed as described (Stock et al., 2007). In brief, the nuclei were sonicated using a Diagenode Bioruptor (Leige, full power 30 s on, 30 s off, in an ice bath for 50 min) to produce fragments of <300 bp. Chromatin (350 μg) was incubated with 5 μg prebound (to Protein A or G magnetic beads, Invitrogen) IgG (Santa Cruz, sc-2025) or antibodies raised to ETS1 (Maier et al., 2003), ETV4 (Abcam, ab860902), ETV5 (Abcam, ab102010), GABPα (Santa Cruz, sc-22810), or ELF1 (Santa Cruz, sc-631) in the presence of 50 μg of BSA, washed, and eluted. Reverse crosslinked DNA was purified with Proteinase K (Glenaxxon) and QIAGEN PCR purification kit. ChIP DNA and input DNA were amplified (WGA2 kit, Sigma), labeled, and hybridized according to the manufacturer's protocol to a 3 × 720,000 probe custom microarray containing specific tiled regions encompassing 8.2 megabases of the mouse genome (Nimblegen). The array platform number is GPL14936 and the GEO accession number for the ChIP data is GSE33997.

Microarray data were analyzed in R/Bioconductor (http://genomebiology.com/2004/5/10/R80) with the Epigenome (PROT43) protocol (http://www.epigenome-noe.net/WWW/researchtools/protocol.php?protid = 43) with the following parameters. The mean signal intensity of the four replicate probes on each array was taken. Loess normalization was used within arrays to correct for the dye bias, and scale normalization was used within the replicates group to control interarray variability. The log enrichment for each group was calculated by subtracting the mean of log2 input intensities from the mean of log2 enriched intensities. Probes were tested for significant enrichment using the significance analysis of microarrays (SAM) technique (Tusher et al., 2001), and the local false discovery rate based on the SAM statistic was calculated using the Locfdr R package (Efron, 2007). A false discovery rate of 0.05 was used as the significance cutoff. The median value of each probe was then calculated from a five-probe rolling window to overcome outliers with values that are very different from their neighboring probes.

## Supplemental Information

Document S1. Figures S1 and S2, Tables S1 and S2, and Supplemental Experimental Procedures
