# Supplementary material for: Opposing Functions of the ETS Factor Family Define Shh Spatial Expression in Limb Buds and Underlie Polydactyly
Source: Dev Cell. 2012 Feb 14;22(2):459–67. doi: 10.1016/j.devcel.2011.12.010 (PMC3314984; doi:10.1016/j.devcel.2011.12.010)
Supplement: Document S1. Figures S1 and S2, Tables S1 and S2, and Supplemental Experimental Procedures [file mmc1.pdf]

Developmental Cell, Volume *22*

Supplemental Information

Opposing Functions of the ETS Factor Family Define *Shh* Spatial Expression in Limb Buds and Underlie Polydactyly

Laura A. Lettice, Iain Williamson, John H. Wiltshire, Silvia Peluso, Paul S. Devenney, Alison E. Hill, Abdelkader Essafi, James Hagman, Richard Mort, Graeme Grimes, Carlo L. DeAngelis, and Robert E. Hill

Supplemental Inventory

Figure S1.
Conservation of the ETS and ETV binding sites predicted in the ZRS, related to Figure 1

Figure S2.
Analysis of the ETV site A protein binding in vitro, and analysis of ETV5 and ETS1 binding to the ZRS in vivo, related to Figure 2

Table S1.
Summary of the number of transgenic animals that were made for each construct, related to Figures 1 and 3

Table S2.
Statistical analysis of the measurements for the Shh expression boundaries, related to Figure 3

Supplemental Experimental Procedures

Supplemental Information

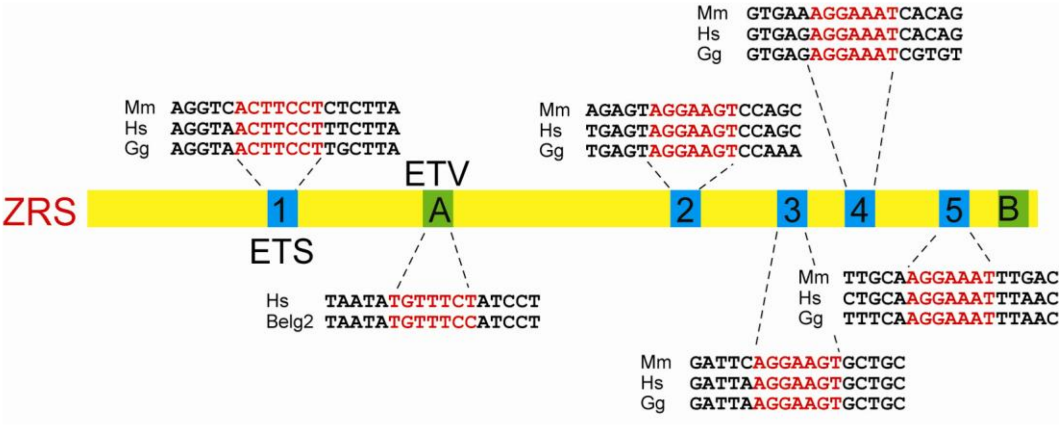

Figure S1. Conservation of the ETS and ETV binding sites predicted in the ZRS, related to Figure 1

Schematic of the ZRS showing the position and surrounding sequences for the five putative ETS (ETS1 And GABPα) and two ETV (ETV4 and ETV5) binding sites identified within the ZRS. ETS binding sites labeled 1-5 and ETV binding sites labeled A and B. Sequence at each of the ETS1 sites is shown, demonstrating the high level of conservation between human (Hs), mouse (Mm) and chicken (Gg). At the ETV A site human wild type (Hs) and mutant (Belg2) sequence is indicated.

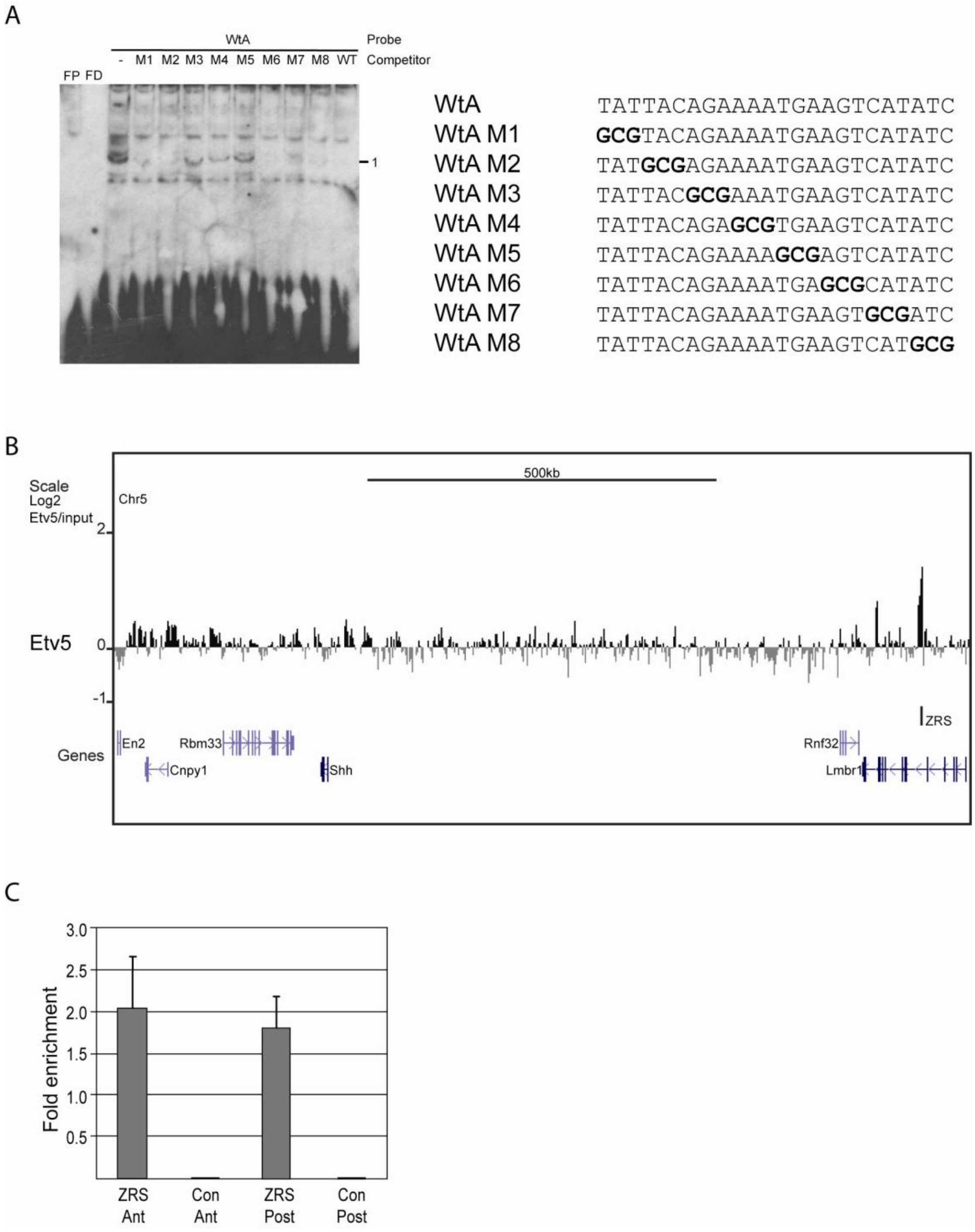

Figure S2. Analysis of the ETV site A protein binding in vitro, and analysis of ETV5 and ETS1 binding to the ZRS in vivo, related to Figure 2

(**A**) ds-Oligos with a consecutive array of 3 bp mutations (M1-M8; the sequence change highlighted in bold) for the ETV site A (WtA) were used in competition assays in 100X excess. The lack of competition in reactions containing M3, M4 and M5

(appearance of band 1) allow the sequence bound by ETV4 to be localized. (**B**) Binding of the ETV5 factor analyzed over the whole Shh region containing the coding sequence, and upstream gene desert. Scale bar shown at top, genes in the region shown at the bottom and position of the ZRS is also shown. (C) ChIP analysis for binding of ETS1 at the endogenous ZRS was confirmed using anterior (Ant) or posterior (Post) halves of E11.5 limb buds. ETS1 at the ZRS (ZRS) was observed in both the anterior and posterior tissue. A control region (Con) (β-actin promoter) was not found to be enriched. Error bars are presented as SEM (n=3).

Table S1. Summary of the number of transgenic animals that were made for each construct, related to Figures 1 and 3.

Table shows the construct, the number of transgenic embryos obtained by genotype, the number that expressed β-gal and where appropriate the number expressing ectopically.

Construct	# of transgenics	# staining in posterior	# staining ectopically (%)
WT (12345)	10	6	
WT+Aus	9	6	5 (83%)
tDel	7	5	
-EtvB	19	6	
-EtvA	8	6	
-EtvA-EtvB	11	10	3 (30%)
+Ts	8	5	
+ETS	11	7	4 (57%)
2345	7	4	
1245	13	7	
345	7	5	
145	4	3	
235	8	7	
125	6	5	
245	8	4	
45	14	5	
5	5	3	
none	6	4	
1	7	4	
3	5	4	
13	8	6	
2345+Aus	9	7	5 (71%)
245+Aus	12	6	2 (33%)
45+Aus	17	13	4 (30%)
+Aus	7	4	0

Table S2. Statistical analysis of the measurements for the Shh expression boundaries, related to Figure 3.

P-values from the Tukey's HSD test of the measurements made for the position of the *Shh* expression boundary for each group of transgenics.

2 3 4 5	1 2 4 5	3 4 5	1 4 5	2 3 5	1 2 5	2 4 5	4 5	5		1	3	1 3	1 2 3 4 5 +Aus	2 3 4 5 +Aus	2 4 5 +Aus	4 5 +Aus	+Aus	ETS sites remaining
1	0.78	1	1	1	1	0.00	0.00	0.00	0.00	0..07	0..19	1	0.00	0.00	1	1	1	1 2 3 4 5
	0.67	1	1	1	1	0.00	0.00	0.00	0.00	0.33	0.55	1	00.00	0.00	1	1	1	2 3 4 5
		0.41	0.18	0.50	0.91	0.00	0.00	0.00	0.00	0.00	0.00	0.23	0.00	0.00	0.51	0.58	0.99	1 2 4 5
			1	1	1	0.00	0.00	0.00	0.00	0.33	0.58	1	0.00	0.00	1	1	1	3 4 5
				1	1	0.00	0.05	0.05	0.00	0.97	0.99	1	0.00	0.00	1	0.99	0.98	1 4 5
					1	0.00	0.00	0.00	0.00	0.11	0..27	1	0.00	0.00	1	1	1	2 3 5
						0.00	0.00	0.00	0.00	0.14	0..29	1	0.00	0.00	1	1	1	1 2 5
							0.79	0.99	0.91	0.01	0.01	0.00	0.00	0.00	0.00	0.00	0.00	2 4 5
								1	0.01	0.91	0.84	0.00	0.00	0.00	0.00	0.00	0.00	4 5
									0.07	0.87	0.79	0.00	0.00	0.00	0.00	0.00	0.00	5
										0.00	0.00	0.00	0.00	0.00	0.00	0.00	0.00	
											1	0.35	0.00	0.00	0.20	0.01	0.02	1
												0.61	0.00	0.00	0..41	0.06	0.08	3
													0.00	0.00	1	1	1.00	1 3
														1	0.00	0.00	0.00	1 2 3 4 5 +Aus
															0.00	0.00	0.00	2 3 4 5 +Aus
																1	1	2 4 5 +Aus
																	1	4 5 +Aus

Supplemental Experimental Procedures

Transgenic constructs

Point mutations and ETS site mutations were made using a QuickChangeII XL site directed mutagenesis kit (Stratagene) while the +Ts and +ETS inserts were generated by PCR. In all cases, the entire ZRS insert was sequenced before use. The oligonucleotides used in the mutagenesis are listed below--

	**Mutagenesis**
AUS	CTTGGCATTTATTACAGAAAA**G**GAAGTCATATCTCACTAACTGTTGC
	GCAACAGTTAGTGAGATATGACTTC**C**TTTTCTGTAATAAATGCCAAG
- Ets Site1	GCACAAAATCTGAGGTCACTT**GAA**CTCTTAATTAGTTGCACTGACCAGG
	CCTGGTCAGTGCAACTAATTAAGAG**TTC**AAGTGACCTCAGATTTTGTGC
- ETS Site 2	CCATCTTAAAGAGAAGAGAGT**TTC**AAGTCCAGCCTGGGACTCC
	GGAGTCCCAGGCTGGACTT**GAA**ACTCTCTTCTCTTTAAGATGG
- ETS Site 3	GTTGGAAATGAGCGATTC**TTC**AAGTGCTGCTTAGTGTTAGTGGC
	GCCACTAACACTAAGCAGCACTT**GAA**GAATCGCTCATTTCCAAC
- ETS Site 4	GTCTGGTTCTGCTGGGTGAA**TTC**AAATCACAGGCAAGAGGAAGG
	CCTTCCTCTTGCCTGTGATTT**GAA**TTCACCCAGCAGAACCAGAC
- ETS Site 5	GGCTCCTGCTGGGAACCTTGCA**TTC**AAATTTGACTTGGGCATG
	CATGCCCAAGTCAAATTT**GAA**TGCAAGGTTCCCAGCAGGAGCC
- ETV Site A	GGAGGAACTAAGATCGTTTTAATAT**CGC**TCTATCCTGTGTCACAGTTTGAG
	CTCAAACTGTGACACAGGATAGA**GCG**ATATTAAAACGATCTTAGTTCCTCC
- ETV Site B	GATCTTGGCATTTATTACAGA**GCG**TGAAGTCATATCTCACTAACTG
	CAGTTAGTGAGATATGACTTCA**CGC**TCTGTAATAAATGCCAAGATC
	**Additional sequence**
+Ts (arm 1)	gatcat**AAGCTT**TACTTTAATGCCTATCTTTG
	GGATCATCAGTGGCAAAAAA**AAAAAAA**AACAAGCAAAAATAATGAAAGAATC

+Ts (arm 2)	GATTCTTTCATTATTTTTGCTTGTT**TTTTTTT**TTTTTTGCCACTGATGATCC
	gatcat**AAGCTTC**ACATAGCAACAGTTAGTGAC
+ETS	gatcat**AAGCTT**TACTTTAATGCCTATCTTTG
(arm 1)	GGATCATCAGTGGCAAAAAA**ACTTCCT**AACAAGCAAAAATAATGAAAGAATC
+ETS	GATTCTTTCATTATTTTTGCTTGTT**AGGAAGT**TTTTTTGCCACTGATGATCC
(arm 2)	gatcat**AAGCTT**CACATAGCAACAGTTAGTGAC

Nuclear extract and electrophoretic mobility shift assays (EMSA)

Nuclear extracts used in the EMSA were prepared from limb tissue using a NE-PER nuclear and cytoplasmic extraction kit (Thermo Scientific).The autopods of fore and hindlimb of E11.5 embryos were removed and either used whole or further dissected into anterior and posterior halves.

EMSA reactions were separated on a pre-run 6% native polyacrylamide gel for 1 hour at 100V, 4°C. Reactions were made up in a total volume of 15μl with 1.5μl 10X EMSA buffer (10mM Tris-Hcl pH7.9, 50mM KCl and 1mM DTT), 1μl of poly dI/dC (Sigma) (1mg/ml) and 6μg of nuclear extract. Samples were incubated for 5 minutes on ice before 20 fmol biotin-labelled probe (oligos end labelled with biotin were purchased from Sigma and annealed) was added and reactions incubated at RT for 20 minutes before loading dye was added and loaded. For depletions, anti-ETS1, anti-GABPα, anti ETS2, anti ELF-1, anti-ETV4 and anti-ETV5 or purified IgG**.** as a control was added along with nuclear extract and incubated for 10 minutes at room temperature prior to addition of the probe. For competition assays, unlabelled probes were added with nuclear extract prior to incubation with the labelled probe. Oligonucleotide pairs used for the EMSA analysis are listed below—

	**EMSA**
WT (AC/AUS)	TATTACAGAAAATGAAGTCATATC
	GATATGACTTCATTTTCTGTAATA
AC	TATTACAGGAAATGAAGTCATATC
	GATATGACTTCATTTCCTGTAATA
AUS	TATTACAGAAAAGGAAGTCATATC
	GATATGACTTCCTTTTCTGTAATA
Et/Pe	GATCTCGAGCAGGAAGTTCGA
	TCGAACTTCCTGCTCGAGATC
Non-specific	CGCTTGATGAGTCAGCCGGAA
	TTCCGGCTGACTCATCAAGCG
ETS Site 1	CTGAGGTCACTTCCTCTCTTAATT
	AATTAAGAGAGGAAGTGACCTCAG
ETS Site 2	GAAGAGAGTAGGAAGTCCAGCCTG
	CAGGCTGGACTTCCTACTCTCTTC
ETS Site 3	GAGCGATTCAGGAAGTGCTGCTTA
	TAAGCAGCACTTCCTGAATCGCTC
ETS Site 4	CTGGGTGAAAGGAAATCACAGGCA
	TGCCTGTGATTTCCTTTCACCCAG
ETS Site 5	AACCTTGCAAGGAAATTTGACTTG
	CAAGTCAAATTTCCTTGCAAGGTT
WT Mut1	GCGTACAGAAAATGAAGTCATATC
	GATATGACTTCATTTTCTGTACGC
WT Mut2	TATGCGAGAAAATGAAGTCATATC
	GATATGACTTCATTTTCTCGCATA
WT Mut3	TATTACGCGAAATGAAGTCATATC
	GATATGACTTCATTTCGCGTAATA

WT Mut4	TATTACAGAGCGTGAAGTCATATC
	GATATGACTTCACGCTCTGTAATA
WT Mut5	TATTACAGAAAAGCGAGTCATATC
	GATATGACTCGCTTTTCTGTAATA
WT Mut6	TATTACAGAAAATGAGCGCATATC
	GATATGCGCTCATTTTCTGTAATA
WT Mut7	TATTACAGAAAATGAAGTGCGATC
	GATCGCACTTCATTTTCTGTAATA
WT Mut8	TATTACAGAAAATGAAGTCATGCG
	CGCATGACTTCATTTTCTGTAATA

Western blot

Nuclear extract was separated on a 12% polyacrylamide SDS gel and protein transferred onto PVDF membranes. The PVDF membrane was incubated in blocking buffer (3% Marvel [dried milk] in TBSTween) before being incubated with primary antibody. After incubation with HRP-labeled secondary antibodies, the membrane was developed with ECL plus reagents (GE Healthcare) as manufacturer's protocol and developed on film or LAS 4010.

qPCR analysis

qPCR was carried out using equal concentrations of input, IgG and Chip DNA using a Sybr Green (Roche) reaction. Enrichment values for ETS1 from extracts from the anterior and the posterior of the llimb bud are presented as fold differences relative to IgG and normalized to input with the formula $2^{[(CtIgG-CtInput)- (CtAb-CtInput)]}$ where Ct values are threshold cycles. The control regions used were the β-actin promoter. All biological replicates were carried out in triplicate unless stated. Primers used for ZRS and the controls are shown below-

**PRIMERS**	**ChIP qPCR**
ZRS	AGAAGAGAGTAGGAAGTCCAGCCT
	GAGCCTTCCTCTTGCCTGTGATTT
Control – β-actin	CCTCGATGCTGACCCTCATCC
	GACACTGCCCCATTCAATGTC
